# Missing data in microrandomized trials: Challenges and opportunities

**DOI:** 10.3758/s13428-025-02885-y

**Published:** 2025-11-14

**Authors:** Shiyu Zhang, John J. Dziak, Lizbeth Benson, Jamie R. T. Yap, Dusti R. Jones, Cho Y. Lam, Lindsey N. Potter, David W. Wetter, Inbal Nahum-Shani

**Affiliations:** 1 Institute for Social Research, University of Michigan, 426 Thompson St, Ann Arbor, Michigan 48104 USA; 2 Huntsman Cancer Institute, Department of Population Health Sciences, University of Utah, Salt Lake City, UT USA

**Keywords:** Microrandomized trials, Just-in time adaptive interventions, Missing data

## Abstract

The vision of leveraging digital technologies to deliver real-time psychological interventions in everyday settings is realized via just-in-time adaptive interventions (JITAI) – an intervention design that guides the use of rapidly changing information about a person’s internal states and contexts to decide whether and how to intervene in daily life. Microrandomized trials (MRTs) were developed as an experimental design to address scientific questions about how to best construct JITAIs, enabling scientists to investigate whether, what type, and under what conditions, intervention delivery can promote behavior change. However, missing data present challenges to the ability of MRTs to inform the development of JITAIs. This article articulates the multiple sources of missing data that can manifest in MRT studies, discusses how such missing data can impact (1) bias, (2) variance, and (3) the future implementation of JITAIs, and discusses strategies for both minimizing missing data in an MRT design and handling missing data when they occur. The overarching goal is to provide a conceptual framework that will guide future investigators in anticipating missing data and making informed decisions to manage them. Throughout, we illustrate concepts using existing data from the Mobile Assistance for Regulating Smoking (MARS) study. MARS (*n* = 99) involved a 10-day MRT that included up to six randomizations per person per day.

**Supplementary Information:**

The online version contains supplementary material available at 10.3758/s13428-025-02885-y.

## Introduction

Over 90% of U.S. adults now own a smartphone (Pew Research Center, 2024). The widespread adoption and reliance on smartphones and other digital technologies have set the stage for the rise of mobile health. Over the past few decades, psychologists have been actively investigating ways to leverage digital technologies to meet the needs of psychological interventions. Digital interventions are now being delivered to help improve quality of life across various domains, such as reducing depressive symptoms (Fu et al., 2020; Moshe et al., 2021), treating anxiety disorders (Pauley et al., 2023; Stefanopoulou et al., 2019), coping stress (Harrer et al., 2024), managing pain (Eather et al., 2022; Palermo et al., 2020), improving psychological well-being (Armaou et al., 2020; Ferrari et al., 2022; Saboor et al., 2024), supporting the reduction and cessation of substance use (Bonfiglio et al., 2022; Fu et al., 2020; Nesvåg & McKay, 2018), and encouraging healthy eating behaviors (Chen et al., 2020; Thomas et al., 2024). Across domains, researchers call for future work to develop a tailored, personalized approach to psychological interventions (Chen et al., 2020; Eather et al., 2022; Fu et al., 2020; Moe-Byrne et al., 2022; Moshe et al., 2021).

*Just-in-time adaptive interventions* (JITAIs) represent an advancement in using digital technologies to deliver psychological interventions in real-time, real-world settings. JITAIs use dynamic information about individuals’ experiences and contexts to decide whether and how to intervene (Nahum-Shani et al., 2018). They are designed to address individuals’ rapidly changing risk of adverse outcomes and to leverage momentary opportunities for positive change (Nahum-Shani et al., 2015). For example, consider a JITAI using a mobile application that sends digital notifications to support smoking cessation (Battalio et al., 2021). Based on sensor-based assessments of an individual’s stress, a decision is made every 2 h about whether to prompt the individual with a stress-regulation strategy. If stress is detected, then a message recommending a stress-regulation exercise is delivered; otherwise, a message is not delivered.

JITAIs closely align with the idea of promoting precision medicine in psychotherapy; they are designed to offer the right amount of support at the right time and place, but no more (Nahum-Shani et al., 2018; Zipfel et al., 2024). This approach to interventions is grounded in theories suggesting that psychological processes are dynamic and fluid, and that people are influenced by a web of environmental forces at any given moment (Cohen et al., 2017). Hence, intervening at the turning points when people are vulnerable to adverse outcomes and receptive to support is essential to facilitating behavioral changes in an effective and cost-efficient way (Cohen et al., 2017; Nahum-Shani et al., 2018).

To assemble effective JITAIs, investigators need empirical knowledge, such as which intervention is more effective in promoting positive changes in the intended near-term outcome and how well different interventions perform under different contexts. Questions regarding the optimal design of JITAIs can be addressed by conducting micro-randomized trials (MRTs) (Liao et al., 2016; Qian, Walton et al., 2022b). The MRT is an experimental design that employs rapid sequential randomizations to different intervention options. This means that it is possible for the same participant to be randomized many times to different intervention options, and the length of the time interval between randomizations may be relatively short (e.g., a few days, hours, or minutes). The MRT is an experimental design that focuses on optimizing interventions; that is, data generated from MRTs are used to produce findings to inform how to construct JITAIs (Walton et al., 2018). The focus of MRTs on optimization differs from standard randomized controlled trials (RCTs), which focus on the *evaluation* of an existing intervention program against a control condition (Collins & Collins, 2018). Although RCTs are critical to determining the overall efficacy of the intervention program, they often do not provide much information on how to improve the intervention. Hence, it has been argued that a logical approach to developing potent digital interventions involves performing optimization trials like MRTs to refine the intervention before evaluating it in RCTs (Szeszulski & Guastaferro, 2024).

There is a growing body of research on MRTs. Since the design was introduced in 2015 (Klasnja et al., 2015), it has been discussed and implemented in over 80 published studies (based on PubMed). The National Institutes of Health has funded at least 34 projects related to MRTs (based on NIH RePORT). These projects span across subfields such as clinical psychology (e.g., interventions for mental health problems), health psychology (e.g., interventions that encourage healthy lifestyles), social psychology (e.g., interventions that consider social influences and group dynamics), and translational research (e.g., applying scientific findings to develop practically effective interventions).

MRTs leverage multiple modes of data collection to yield rich and granular data for each participant. For example, participants’ experiences and behaviors can be assessed following each randomization via ecological momentary assessment (EMA) (Shiffman et al., 2008; Smyth & Stone, 2003), which enables analyses of near-term benefits of delivering one intervention option over another. Time-varying variables are also used to describe the contexts in which randomizations occurred (e.g., sensor data collected on smartphones are used to classify minutes as “probably stressed” or “probably not stressed”) (Battalio et al., 2021). This information can be used to investigate the conditions in which one intervention option is more beneficial than another (e.g., is messaging more effective during “probably stressed” or “probably not stressed” moments). An extensive set of static, person-level features is collected at the baseline assessment, which may be used to determine how individual characteristics and behavioral histories affect the efficacy of different intervention options. Given the intensive, multi-modal nature of data collection in MRTs, missing data are inevitable.

Missing MRT data limits researchers’ ability to answer scientific questions. First, missing data reduce the number of participants and randomization occasions available for analysis, thus undermining statistical power for detecting intervention effects. Unlike randomized trials that evaluate end-of-study outcomes, intervention effects in an MRT are estimated based on intensive longitudinal outcome data, so it is highly likely that most participants will have at least some missing data on the outcome variable.

Second, missing data can undermine the accuracy of the estimated intervention effects if the probability of missingness varies among participants and across time. For example, the intervention effect may be biased if missing data are more prevalent in specific subpopulations or circumstances, and if the intervention works differently for those individuals or in those circumstances. Suppose that when participants are experiencing stress, they are less inclined to follow the suggested intervention and also less likely to report their outcome via an EMA. The observed data may overrepresent moments when the suggested interventions are well received, leading to an overestimated intervention effect.

Third, in many realistic situations, missing data in MRTs may imply that there will also be missing data in future JITAIs. MRT data can be analyzed to provide practical insights on how to handle the missingness in JITAIs. For example, if information on participants’ stress levels is sometimes missing during an MRT because participants forget to wear the sensor for detecting stress, then most likely this information will also sometimes be missing during a future JITAI for the same reason. A JITAI should incorporate protocolized rules about how to act during circumstances when information about participants is missing, and these rules may be empirically developed by analyzing missing data in MRTs.

The scientific and practical implications of missing data in MRTs have seldom been discussed in prior research (for exceptions, see Kondo & Oba, 2024; Seewald et al., 2019), limiting the ability of researchers to plan strategies for minimizing and addressing missing data. To close this gap, this paper maps out the multiple sources of missing data in MRTs and their distinct implications for the bias (i.e., the systematic deviation from the true intervention effect) and precision (i.e., the variance of the estimated intervention effect) of estimated intervention effects, and their implications for the design of JITAIs in practice. Our goal is to provide guidelines for investigators to minimize and address various types of missing data in MRTs.

In the following, we begin by defining JITAIs and introducing an MRT study, the Mobile Assistance for Regulating Smoking (MARS; Nahum-Shani, Potter et al., 2021a) study, which we use as an illustrative example (Section "Key components of JITAIs and an example of MRT"). Next, we identify and define five types of variables typically collected in MRTs (Section "Missing data in different types of MRT variables"): proximal outcome (3.1), covariates (3.2), embedded tailoring variables that do (3.3) and that do not restrict randomizations (3.4), and candidate tailoring variables (3.5). For each type of variable, we discuss (a) how missing data can affect the *point estimates* (i.e., increase bias) of the intervention effects and their interpretation, (b) whether and how the specific source of missingness may reduce statistical precision (i.e., increase *variance*) of the estimated intervention effects, and c) how missing data may be *practically* related to the implementation of future JITAIs. Finally, in Section "Multiple imputation in MRT", we discuss challenges pertaining to performing multiple imputation (MI) in MRTs, explain challenges for implementing MI in MRTs given their unique design, and discuss why MI may not always be suitable for handling missing data in MRTs.

## Key components of JITAIs and an example of MRT

### Components of JITAIs

JITAIs include the following components: distal outcomes, proximal outcomes, intervention options, tailoring variables, decision points, and decision rules (Nahum-Shani et al., 2023; Qian, Walton et al., 2022b). Definitions and examples from the MARS study (described in more detail below) are shown in Table 1.

**Table 1:** Definitions and examples of JITAI components

Components	Definitions	Examples
Distal outcome	The ultimate long-term goal of the JITAI	Smoking cessation defined as 7 consecutive days of abstinence by week 12
Proximal outcome	Short-term behaviors or experiences that the JITAI is intended to impact. Desired changes in the proximal outcomes are expected to facilitate desired changes in the distal outcome	Engagement with self-regulatory strategies in the past hour
Intervention options	Different types of interventions, intensities, delivery modalities, or tactics under consideration	Three intervention options: – Prompting with a low-effort self-regulatory strategy – Prompting with a more effortful self-regulatory strategy – No prompting
Tailoring variables	Information used to determine the appropriate intervention option for the participant	Participants’ self-reported stress level
Decision points	Time points when an intervention option is selected based on available information about the participant	Every 2 h during participants’ waking hours
Decision rule	A set of “if-then” statements that connect tailoring variables to intervention options. They specify how the information about participants’ contexts/experiences should be used to select the intervention option for delivery	At each decision point: IF stress level = high, THEN intervention option = prompting with a self-regulatory strategy ELSE, intervention option = no prompting

### MRT example: Mobile Assistance for Regulating Smoking (MARS)

**Fig. 1:**
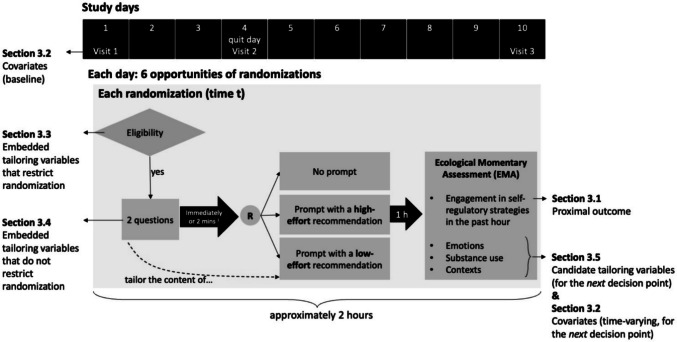
Study design of MARS. *Note* ^1^ The randomization was implemented either immediately after participants responded to the two questions or two minutes after the two questions were administered

MARS includes a 10-day MRT that investigated the effect of delivering digital prompts to encourage engagement with self-regulatory strategies to support smoking cessation (Nahum-Shani, Potter et al., 2021a). Figure 1 briefly summarizes the MARS study design and maps out how the collected data serve different purposes for the intervention and research evaluation. More details about the MARS design can be found in Potter et al. (Under Review).

Participants were asked to use a mobile app, which housed all intervention content. Each study day contained six decision points, separated by approximately 2-h intervals. Within each 2-h interval, *eligibility* for randomization was checked. A decision point was randomized only if it was deemed eligible for randomization, meaning that all experimentally tested intervention options were considered appropriate at that time. In MARS, eligible decision points were defined as those in which the participant was not driving and had not turned on privacy mode on their phone. If a decision point was eligible, two questions were administered asking about the person’s negative affect (yes/no) and cigarette availability (yes/no). If participants completed these questions, they were immediately randomized to one of three intervention options; otherwise, they were randomized 2 min later. The three intervention options include (1) no prompt, (2) prompt recommending a low-effort self-regulatory strategy, and (3) prompt recommending a more effortful self-regulatory strategy. The low-effort prompts were tailored based on the participant’s answers to the two questions (i.e., prompts were drawn from four sets of messages crafted based on yes/no responses to the two questions). If the participant did not answer the questions, the low-effort prompts were drawn from the set of messages for cases where participants reported no negative affect and no cigarette availability. The more effortful prompts were not tailored; they recommended one of five randomly selected cognitive or mindfulness-based exercises that the participant could access in the mobile app. One hour following each randomization, participants were invited to complete an EMA, which included items pertaining to participants’ engagement with self-regulatory strategies in the past hour, as well as their emotions, substance use, and intrapersonal and contextual information. If a decision point was not eligible for microrandomization, then no EMA was delivered by design.

In addition to using the mobile app, participants were also asked to complete three in-person visits on days 1, 4, and 10. During these visits, participants completed survey questionnaires measuring constructs such as their sociodemographic characteristics, as well as risk and protective factors related to tobacco use.

MARS had three prespecified analytic aims. The primary aim was to estimate the average proximal effect of prompting (vs. no prompting) on participants’ self-reported engagement with self-regulatory strategies in the next hour. The secondary aim of MARS was to estimate the average proximal effect of prompting with a low-effort strategy, compared to prompting with a more effortful strategy, on self-reported engagement with self-regulatory strategies in the next hour. Exploratory analyses examined whether the effects of promptings specified in the primary and secondary aims were moderated by participants’ contexts and experiences. All analyses used data from 99 participants between study days 2–9 (only days 2–9 were included because very few randomizations occurred on days 1 and 10; several sensitivity analyses were also conducted to assess robustness of results). This corresponded to a total of 4752 decision points (99 participants × 8 days × 6 decision points per day). The MARS results are reported in Potter et al. (Under Review).

### Missing data in different types of MRT variables

In this section, we articulate how missing data in different types of MRT variables have different implications for a) the *point estimates* (bias) of the intervention effects, b) the *precision* (variance) of the intervention effect estimates, and c) *future JITAIs in practice*. Table 2 summarizes variable definitions, shows their correspondence to the MARS example, provides question item examples, and illustrates the temporal alignment of these variables in the analysis. Appendix 1 provides step-by-step instructions using R code and artificial data to demonstrate the analyses and methods.

**Table 2:** Definitions and examples of key MRT variables

	*MARS example*		
MRT variables	Construct	Variable operationalization	Temporal alignment (i.e., How variables are measured at different points in time and how they are related in the analytic model)
Intervention Indicator ^1^ *Definition:* An indicator for the intervention assignment at each decision point	Whether/how participants are prompted	App-generated. Categorical variable: - no prompt - prompt recommending a low-effort self-regulatory strategy - prompt recommending a more effortful self-regulatory strategy	time $t$ where $t$ indicates decision points
Proximal Outcome – Section 3.1 *Definition:* Near-term behaviors or experiences that the intervention options are intended to change. It is analyzed as the outcome variable in an MRT, where the goal is to assess the effect of intervention options on the proximal outcome	Engagement with self-regulatory strategies in the past hour, measured following *each* (eligible) randomization	Self-reported - Q1 Think about the most recent tip or activity recommended by MARS. Did you use it in the last hour? - Q2 In the last hour, did you use any other tip or activity recommended by MARS? - Q3 In the last hour, did you use any other tip or activity not included in MARS? Binary variable summarizing Q1-3: - Used any self-regulatory activity - Did not use any self-regulatory activity	(shortly) after time $t$ The proximal outcome is often measured shortly after each time-$t$ randomization, before the next decision point at time $t+1$. In MARS, the proximal outcome was measured one hour after each randomization (while the next decision point, within the same day, was approximately 2 h after).
Covariates – Section 3.2 *Definition:* Variables that are not of primary scientific interest but are included in the analyses as additional predictors of the proximal outcome for the purpose of improving the precision of the estimated intervention effect. They must be assessed prior to each randomization to avoid conditioning on variables affected by the randomization	E.g., Baseline: age E.g., Time-varying: Proximal outcome at the *previous* time point	E.g., Proximal outcome at the *previous* time point: This binary variable is operationalized the same way as the proximal outcome described in the above cell, but it is measured at an earlier time point	before time $t$ In MARS, baseline covariates were measured at the beginning of the study, and time-varying covariates were measured via the EMA administered at $t-1$
Embedded Tailoring Variable that Restricts Randomization (i.e., Eligibility for Randomizations)– Section 3.3 *Definition:* Variables that are integrated into the MRT by design and restrict the randomizations to conditions when all intervention options are deemed appropriate ethically, clinically, or practically	Driving a car and phone on privacy mode	Passive data generated by the smartphone sensor and operation system Binary variables: Yes/No	before time $t$ In MARS, this information was collected less than two minutes before each randomization at time $t$
Embedded Tailoring Variables that do not Restrict Randomization – Section 3.4 *Definition:* Tailoring variables that are integrated into the MRT by design but do not restrict randomizations	The two questions about participants’ negative affect and cigarette availability	Self-reported - “Cigarettes are available to me.” - “Are you experiencing a negative emotion?” Binary variables: Yes/No	before time $t$ In MARS, this information was collected from participants two minutes before each randomization at time $t$
Candidate tailoring variables – Section 3.5 *Definition:* Information measured during the trial about the person’s state and context that investigators would like to study as potential moderators of the intervention effects and consider as tailoring variables in a future JITAI	E.g., risky behaviors	Self-reported E.g., “Since the last assessment, did you use any of the following?” Categorical variable: none/cigarettes/e-cigarettes/cigars/smokeless tobacco/marijuana/alcohol	before time $t$ In MARS, candidate tailoring variables were measured via the EMA administered at time $t-1$

*Note* ^1^ Not discussed in this paper. Intervention assignment indicator is automatically generated by the app at each randomized occasion; it usually has no missing data unless there are app-related malfunctions

### Proximal outcome

The primary outcome in an MRT is a proximal outcome representing near-term behaviors or experiences that the intervention options are intended to change. In MARS, the proximal outcome of primary interest is a binary indicator of participants’ engagement with self-regulatory strategies during the one hour following randomization (1 = any engagement; 0 = no engagement).

A complete-case analysis remains the most common approach to handling missing data in MRT analyses. In a complete-case analysis, decision points with missing values in any of the variables in the data analytic model, including the proximal outcome, covariates (see Section “Covariates”), or candidate moderators (see Section “Candidate Tailoring Variables to Inform Future JITAIs”), are excluded; intervention effects are estimated only based on decision points that have complete data on all variables in the data analytic model.

#### Implications for point estimates (bias)

Excluding decision points with missing data in the proximal outcome violates the principle of intention-to-treat analysis (Alshurafa et al., 2012) and can compromise balance in both measured and unmeasured confounding factors established by randomization. As a result, intervention effects estimated using only complete proximal outcome data may be biased. Although it is not possible to determine the degree of bias in the estimated intervention effect from the observed data alone, evidence of potential bias can be obtained by inspecting patterns of missingness. Specifically, investigators can estimate the association between participant characteristics and contexts, and the prevalence of missing data. Strong associations between missingness rates and certain conditions may suggest that missing-data prevalence depends on those conditions; in such cases, MRT results under a complete-case analysis should be interpreted with caution.

**Fig. 2:**
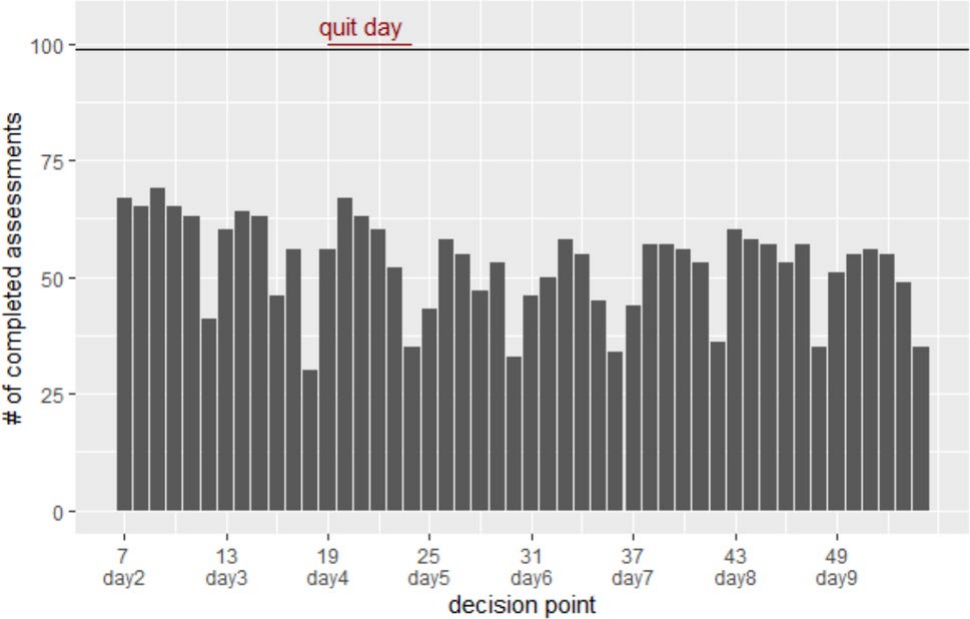
The proportion of completed outcome assessment by decision point

To illustrate one type of analysis that can be done to investigate missing data patterns, we examined how the amount of self-reported data in the primary proximal outcome changed over time and varied across participants with different sociodemographic characteristics using MARS data. First, we plotted the proportion of completed outcome assessments by the 99 participants at each decision point in Fig. 2 (proportion = number of completed outcome assessments/number of decision points eligible for randomization). Figure 2 demonstrates that the completion rate was consistently low in the last 2-h interval of each study day, so the proximal outcome of prompts in the later part of the day was underrepresented in the analytic data. If the intervention’s effectiveness varied toward the end of the day, the estimated intervention effect based on complete outcome data might be biased.

Next, we found that the amount of missing data varied substantially across participants. To investigate whether there were systematic patterns in the occurrence of missingness, we fitted separate linear regression models to estimate associations between participants’ sociodemographic characteristics at baseline (i.e., age, sex, race/ethnicity, income, marital status, and tobacco history) and the proportion of missingness in the proximal outcome for each participant. There was some evidence indicating that participants who were Latino (the reference category was non-Latino White) were more prone to having missing values in the primary proximal outcome. In contrast, participants with histories of heavier tobacco use tended to have fewer missing values in the proximal outcome (compared to those with histories of lighter tobacco use), which might reflect their elevated motivation to participate in the smoking cessation intervention. If prompting had different effects among these subpopulations, then the missingness in the proximal outcome might introduce bias into the estimated effect of prompting on subsequent engagement in self-regulatory strategies.

These analyses suggest ways to investigate possible bias in the estimated intervention effects. If no significant association is detected between important observed variables and missingness, investigators may be somewhat more confident that missing data in the proximal outcome do not lead to misleading conclusions about the intervention effects. However, it is important to recognize that this does not eliminate the possibility of bias. The estimated intervention effects may still be biased if, for example, the missing pattern is related to the proximal outcome itself (i.e., missing not at random missing data mechanism; Rubin, 1976), but this cannot be detected from observed data.

#### Implications for variance (precision)

Under a complete-case analysis of MRT data, intervention effects are estimated using only decision points with non-missing proximal outcome values. In MARS, the missing rate of the primary proximal outcome was 35% (1337 out of 3860 decision points eligible for randomization). This missingness reduced the number of decision points available for analyses and, in turn, reduced the precision of the estimated intervention effects relative to the precision that could have been obtained if no data had been missing.

### Covariates

Covariates, in this context, are variables that are not of primary scientific interest but are included in the analyses as additional predictors of the proximal outcome for the purpose of improving the precision of the estimated intervention effect (i.e., reducing standard errors) (Qian, Cohn et al., 2022a, Qian, Walton et al., 2022b; Shi et al., 2025). For example, to the extent that age is associated with self-regulatory engagement, including age as a covariate in the primary aim analysis of MARS may reduce the standard error for the estimated effect of prompting (vs. no prompting) on self-regulatory engagement. In MRTs, covariates may include static individual characteristics measured at the beginning of the study and/or time-varying experiences/behaviors measured up to and at each decision point.

In randomized trials, the considerations that go into the selection and interpretation of covariates are different from those in observational studies. First, coefficients of the covariates are not of primary scientific interest, as the goal of randomized trials is almost always to estimate effects related to the randomized interventions. Covariates are included primarily to improve the precision of the estimated intervention effects. Second, and relatedly, covariates are not mandatory for obtaining an unbiased estimate of the intervention effect (Boruvka et al., 2018; Qian, Cohn et al., 2022a, Qian, Walton et al., 2022b; Shi et al., 2025), as the randomization procedure is already designed to balance distributions of covariates across experimental conditions. These considerations arguably lead to a unique perspective on the management of missing data on covariates in randomized trials—that is, the strategies for handling missingness in covariates primarily need to ensure that the *estimated intervention effect* is unbiased and precise; whether the *estimated coefficients of covariates* are also unbiased and precise is of secondary importance. In fact, the R package MRTAnalysis that is used to estimate causal effects for MRTs does not output coefficients of covariates by default (Qian et al., 2023).

With these considerations in mind, we discuss the implications of a complete-case analysis and a promising alternative, the missing-indicator method. The missing indicator method is a practical and efficient strategy for handling missing covariate data when estimating the average intervention effect in standard randomized controlled trials (Groenwold et al., 2012; Kamat & Reiter, 2021; Sullivan et al., 2018; White & Thompson, 2005; Zhao & Ding, 2024). It refers to treating the missing values of a covariate as their own category and creating a separate indicator to denote the missingness. For example, using the missing-indicator method on a variable “income” involves replacing the missing income values with a constant (such as a grand mean; the choice of constant only affects the intercept term in the regression model used to analyze the data), creating a new binary variable to indicate the missingness (e.g., 0 for reported income and 1 for missing income), and then including both the constant-imputed income and the missing indicator as a covariate pair in subsequent analyses. Appendix 2 provides a detailed explanation of this method. In randomized-controlled trials, a simple approach like the missing-indicator method has the potential to address the impact of missing covariate data – helping to avoid bias and inflated variance (Groenwold et al., 2012; Kamat & Reiter, 2021; Sullivan et al., 2018; White & Thompson, 2005; Zhao & Ding, 2024). In MRTs, at least for baseline covariates, the missing-indicator method may similarly be useful, with additional research needed to validate its usefulness for managing missingness in time-varying covariates.

#### Implications for point estimates (bias)

Missingness in covariates may bias the estimated average intervention effects if a complete-case analysis is performed. By excluding decision points with missingness in covariates, the principle of intention-to-treat analysis is violated. The estimated intervention effect is biased to the extent that the effectiveness of the intervention options differs between participants and/or decision points with missing covariates and those with complete covariates. In many cases, it is possible to anticipate the level of missingness in commonly used covariates. For example, there is evidence indicating that questions on sensitive topics like “income” tend to contain a substantial amount of missing data, and this missingness may be especially common among those with very high or low income (Frick & Grabka, 2014; Tourangeau & Yan, 2007). Thus, if one decides to perform a complete case analysis, it is advisable to avoid covariates with a high proportion of missing values. Importantly, the selection of covariates must be pre-specified before the MRT data are collected or analyzed to ensure the integrity of the results.

Other than avoiding covariates that are expected to be partly missing, the impacts of missing covariates on the bias of the intervention effect may also be mitigated by alternative model specifications like the missing-indicator method. Although the missing-indicator method is considered invalid for handling missing data in observational studies (Graham, 2009; Schafer & Graham, 2002), it can be valid for estimating the average intervention effects for randomized trials. The theoretical foundation of this method was originally established in the context of standard randomized controlled trials (Groenwold et al., 2012; Kamat & Reiter, 2021; Sullivan et al., 2018; White & Thompson, 2005; Zhao & Ding, 2024); similar reasoning may support its application for addressing missing covariates in MRTs when estimating the average intervention effect. Specifically, the validity (unbiasedness) of the estimated intervention effect is rooted in the randomization procedure. Bias in the intervention effect due to missing covariates can be avoided as long as the missing data strategy keeps the intervention assignment independent of covariates and also retains all participants and decision points in the analysis. A simple model specification technique like the missing-indicator method achieves precisely these goals. However, it is critical to emphasize that, when the missing-indicator method is used, only the estimated average intervention effect is unbiased; the estimated coefficients for covariates can be biased and should not be interpreted. This is because covariates are still mutually correlated; the use of missing indicators distorts the estimated covariance structure among covariates and the outcome, leading to overestimation or underestimation of the prognostic effects of covariates on the outcome.

#### Implications for variance (precision)

Missing data reduce the sample size under a complete-case analysis and impact the precision of the model estimates. The precision loss due to missing data in covariates can diminish and even cancel out the precision gains from including the covariates in the model. For example, consider the list of covariates that were pre-specified for MARS analyses (e.g., age, sex, marital status, income, tobacco use history, and the primary proximal outcome measured at the most recent eligible decision point); 32% (799/2523) of decision points contain missing values in at least one of the covariates. Using these covariates under a complete-case analysis would substantially reduce the number of decision points available for estimating the intervention effects. It is very possible that including covariates with missing values can counter the purpose of improving estimation efficiency.

The missing-indicator method is a simple approach that leverages the predictive power of the incomplete information in the missing covariates (i.e., does not have to drop columns with missing data) and still retains the analytic sample size (i.e., also does not drop rows). It is thought to have good asymptotic efficiency (Zhao & Ding, 2024). However, this efficiency is not guaranteed in small samples because a missing indicator is created for each covariate that contains missing values, resulting in the number of predictors increasing quickly, with a higher risk of overfitting (White & Thompson, 2005; Zhao & Ding, 2024).

### Embedded tailoring variables that restrict randomizations

Embedded tailoring variables are those integrated into the MRT by design. In an MRT, a priori knowledge based on previous studies, theories, or experiences can be used to restrict the randomizations—namely, randomization to intervention options occurs only under conditions in which these options are deemed appropriate based on scientific, clinical, practical, and ethical considerations (Qian, Walton et al., 2022b). *Eligibility status* of each decision point is a binary indicator that differentiates between decision points that meet the pre-specified conditions and thus are considered eligible for randomization, and those that do not meet these conditions (the term “availability” is also used; e.g., Boruvka et al., 2018; Klasnja et al., 2015; Seewald et al., 2019). For example, in MARS, a decision point was eligible for randomization if the person was not driving a car and did not turn on the privacy mode on the phone. In other words, eligibility status is a form of embedded tailoring variables that determine whether decision points are randomized.

When the information needed to determine the eligibility of a decision point is missing during the trial (e.g., if driving and privacy mode data are missing in the case of MARS), it is not clear whether the decision point should be randomized. Investigators can plan the MRT to handle this situation. One option is to treat decision points with missing eligibility information as eligible and randomize them; the other option is to treat them as ineligible and not randomize them. This decision should be driven by considerations such as the effort and burden required from participants to engage with the intervention options, the financial cost associated with delivering them (e.g., if an intervention option involves incentives), and the anticipated likelihood that decision points with missing eligibility information consist of occasions that actually should not be randomized (e.g., the likelihood that participants are actually driving when their driving status is missing). In MARS, investigators decided to randomize the decision points when the data on driving and privacy mode were missing.

#### Implications for point estimates (bias)

In MRTs where the randomizations are restricted to eligible decision points, the estimated intervention effect is conditional and should be conceptualized as the difference in the proximal outcome between different intervention options delivered *at decision points eligible for randomizations* (Boruvka et al., 2018; Qian, Cohn et al., 2022a). Thus, whether decision points with missing eligibility information are considered eligible and subsequently randomized changes the meaning and value of the intervention effect that one is trying to estimate. Specifically, when investigators decide to randomize decision points with missing eligibility information, the average intervention effect is a composite of the effect at decision points that are classified as eligible based on complete information and the effect at decision points that contain no eligibility information. Suppose there are reasons to believe that intervention options delivered at ineligible decision points are mostly ineffective (e.g., participants do not interact with phone messages sent when they are unable to use their phones, so sending messages cannot affect the proximal outcome). To the extent that the decision points with missing eligibility information consist of occasions that should have been classified as ineligible had the eligibility information been available, the estimated intervention effect may be attenuated, compared to the estimated effect under the alternative possible decision to not randomize decision points with missing eligibility information.

In the case when decision points with missing eligibility information are randomized, investigators may perform a sensitivity analysis to evaluate how the decision to randomize these decision points affects the estimated intervention effect. This can be done by moderation analyses where the missingness status of the eligibility information (i.e., a binary variable indicating that the eligibility information is observed vs. missing) is tested as a potential moderator of the intervention effect. A non-significant interaction effect suggests that it may be reasonable to treat the decision points with missing eligibility information as eligible. However, if a significant interaction effect was observed, it implies that the intervention effect varied between decision points with complete eligibility information and those with missing eligibility information. The two scenarios may need to be represented by different intervention effects and addressed separately in future JITAIs.

#### Implications for variance (precision)

Tailoring variables that restrict randomization (i.e., eligibility status of decision points) are part of the MRT design; thus, how missing information in these variables is managed can substantially change the number of possible data points that will be generated during the MRT. Naturally, this affects the number of decision points (number of rows) that can be used to estimate the intervention effect. For example, in MARS, among the total possible 4752 decision points, 58% had complete information on whether participants were driving a car and whether their phone was on privacy mode (19% and 40% classified as ineligible and eligible, respectively), while the remaining 42% were missing either driving or privacy mode data. Since MARS investigators decided to treat decision points with missing data on driving or privacy mode as eligible, 40% + 42% of decision points were randomized for estimating the intervention effect. As mentioned previously, the other option could have been to treat decision points with missing data on driving or privacy mode as ineligible for randomization. In that case, the number of randomization occasions that could be used to estimate the intervention effect would be halved (i.e., only 40% of decision points would have been randomized). Given this large difference in the number of possible data points, the intervention effect estimated in the former design (randomizing when eligibility information is missing) most likely has a smaller standard error than that estimated in the latter design (not randomizing when eligibility information is missing). However, the values of these two standard errors should not be directly compared because the meaning of the intervention effect has already been changed under the two design options.

#### Future JITAIs in practice

Setting a decision point as ineligible for randomization in MRTs (e.g., due to phone privacy mode or driving) means that investigators have already decided how to act on these occasions (e.g., do not disturb the participant), so no experimentation is needed to answer questions about the delivery of intervention options in those conditions. As such, if an MRT is designed not to randomize decision points with missing eligibility information, it implies that the future JITAI will treat these decision points the same way they are treated in the MRT. On the other hand, if an MRT is designed to randomize decision points with missing eligibility information, as in MARS, it means that all the experimentally tested intervention options are considered feasible when eligibility information is missing, and so which option should be delivered in a future JITAI depends on the results of the trial. For example, by investigating whether missing (vs. complete) eligibility information moderates the intervention effect, investigators can ascertain whether the intervention effect varies between occasions with missing eligibility information and occasions with complete information. If the results indicate that delivering a prompt is not effective when eligibility information is missing, then the future JITAI can refrain from delivering a prompt under these conditions.

### Embedded tailoring variables that do not restrict randomizations

A priori knowledge about tailoring variables can also be incorporated into MRTs without restricting randomization. For example, based on existing evidence and practical considerations, the content of the intervention options can be tailored based on a person’s current experiences and contexts. In this setting, handling missing data means that investigators should specify a course of action in case data on the embedded tailoring variable are missing (e.g., specify a fallback version of the intervention option that does not depend on input information from participants). In MARS, participants’ self-reported negative affect and cigarette availability served as embedded tailoring variables that did not restrict randomizations (i.e., the self-report was not used to decide whether a prompt would be delivered, but only the wording of the low-effort prompts). If a participant reported negative emotions immediately prior to a given decision point, a prompt tailored to this status might be something like: “When you are feeling stressed, leave if cigarettes are available or drink a tall glass of water. You are in control!” However, if a participant did not answer the questions about negative affect and cigarette availability, a generically worded prompt such as “Be proud of the work you are doing to change your life! Create a list of rewards you can give yourself for staying quit” was sent instead.

#### Implications for point estimates (bias)

Missing data in such embedded tailoring variables may have implications on the overall effectiveness of the intervention and, in turn, lead to different values and interpretations of intervention effects. In the MARS example, low-effort prompts that were not tailored could be less effective than those tailored to participants’ self-reported emotions/contexts. A high proportion of missing data in negative affect/cigarette availability would result in a higher proportion of non-tailored prompts (instead of tailored prompts) being delivered, which may weaken the effect of prompting with low-effort strategies in encouraging engagement with self-regulatory strategies. Therefore, the amount of missing data and the strategy for handling missing data of embedded tailoring variables provide an important context for interpreting the estimated intervention effect. We suggest that investigators protocolize the strategy and report the missing data rate so that the findings can be clearly interpreted and replicated in future studies.

#### Implications for variance (precision)

Unlike the other types of MRT variables, missing data in embedded tailoring variables that do not restrict randomizations do *not* cause incompleteness in the dataset for MRT analyses. That is, this form of missingness does not reduce the number of decision points (rows) available for estimating intervention effects. In the MARS example, all 997 decision points randomized to the low-effort condition are used to estimate the intention-to-treat effect of prompting with low-effort recommendations. This is true even though 37% of decision points (366/997) had missing values in participants’ self-reported negative affect and cigarette availability, as this missingness only affected the specific content of the messages delivered.

#### Future JITAIs in practice

When tailoring variables that do not restrict randomizations are embedded in an MRT, it means that investigators plan to tailor the future JITAI this way. The plan includes both how to intervene when tailoring variables are observed and how to act in case tailoring variables are missing. Yet, before integrating such planned tailoring in an optimized JITAI, investigators may want to evaluate whether tailoring an intervention option indeed enhances the intervention option’s effectiveness as expected, in comparison to not tailoring. One way to explore this is to examine whether the missingness (vs. completeness) of tailoring variables moderates the intervention effect (this analysis is observational, not causal). In the MARS example, if the tailoring based on participants’ self-reported current emotions/contexts worked as intended, the tailored messages should yield a stronger intervention effect than the non-tailored messages. If this pattern is not observed, it suggests that prompts seem to work about as well without content tailoring as with content tailoring. Perhaps the content tailoring was not needed or not done well, and investigators may want to rethink and refine this use of tailoring variables. Importantly, because the decision to tailor (vs. not tailor) is not randomized in this scenario, findings from this analysis are associational rather than causal.

In a scenario when embedded tailoring variables are based on participants’ self-reports, as in MARS, it is possible that missingness represents a situation in which a participant is not fully available to engage with requests. For example, if a participant is too busy, distracted, or tired to answer a brief questionnaire intended for tailoring a prompt (i.e., unavailable to report negative affect and cigarette availability), then the participant may also be unlikely to pay much attention to the prompt itself (i.e., unavailable to engage with the prompt two minutes later). Ironically, the observability versus unobservability of certain information about a participant’s state can still be a clue to this state, becoming a kind of indirect information rather than missing information in the classic sense (i.e., missingness of negative affect/cigarette availability hints at the likelihood of prompt engagement). In the design of JITAI, this means that missing data on participants’ current status/contexts may still be valuable for determining the timing for delivering intervention options effectively.

### Candidate tailoring variables to inform future JITAIs

Candidate tailoring variables are measurements collected during the MRT about the participant’s state and context, which investigators consider and investigate their usefulness as tailoring variables in a future JITAI. After the study concludes, these variables are examined as possible moderators of the intervention effects by testing their interactions with the random intervention assignment. Plausible candidates are typically those involved in cross-over interactions, which occur when the direction of the intervention effect differs between levels of the moderator (Gunter et al., 2011). For example, if the intervention is effective at certain levels of a particular observed variable *X* and is non-effective or even detrimental at other levels, this would be very informative for prescribing the delivery of intervention, suggesting the inclusion of variable *X* as a tailoring variable in future JITAIs.

In MARS, examples of candidate tailoring variables include emotional experiences (feeling ashamed, guilty, happy, and stressed), mental capacity (self-regulation), and risky behaviors (nicotine use, marijuana use, and alcohol consumption), operationalized as measurements at the previous decision point. For MARS participants, EMAs at the previous decision point represent the most recent information available for characterizing their status at the current decision point; of course, investigators may consider other operationalizations such as the average scores on measurements collected in the past 24 h. Additionally, a different MRT design could collect the information about candidate tailoring variables in the EMA just before the randomization at each decision point (for MARS, this corresponds to expanding the two-question survey administered two minutes before each randomization). This approach would eliminate the need to rely only on information collected from the previous decision point, which might no longer be relevant for guiding intervention delivery at the current decision point if decision points are placed far apart. However, the disadvantage is that adding more assessments increases the burden on participants, which can increase missingness.

#### Implications for point estimates (bias)

Missing data in candidate tailoring variables should only affect exploratory results in most MRTs, because the variables are studied as potential moderators of intervention effects and such analyses are almost always specified as exploratory rather than primary aims. The common approach for handling missing data in this setting is still a complete-case analysis. Excluding decision points with missing values in candidate tailoring variables may cause the composition of the analytical sample to deviate from that of the recruited sample and, in turn, introduce biases into the estimated interaction effects (where “bias” refers to deviations from the estimates that would be obtained using the full recruited sample with complete data). For example, if a potential moderator is defined as the measurement at the previous decision point, then the estimated interaction effect tends to be based more on data from individuals who participate continuously and less on data from individuals who participate intermittently. In an extreme scenario, the analysis will include all data from someone who participates for one day and then drops out of the study, but exclude all data from another person who participates in every second decision point. To the extent that the intervention effect varies across individuals participating on different schedules, missing data on this potential moderator may introduce biases into model results.

#### Implications for variance (precision)

**Fig. 3:**
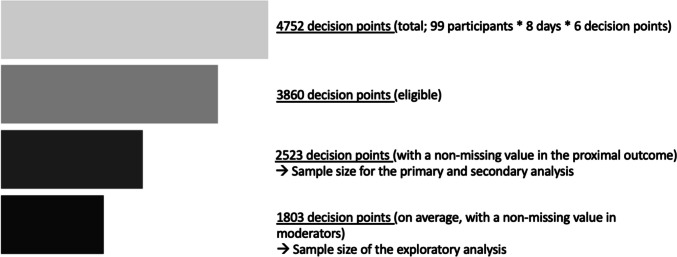
Changes in the number of decision points available for analyses due to different sources of missingness. These changes in sample size affect both the biases and precision of the estimated intervention effects

Missing data in candidate tailoring variables reduce the number of decision points available for analysis and therefore impact the precision of the estimated interaction effects. For the list of potential moderators analyzed in MARS, on average, 28% of decision points contained missing values (out of the 2523 decision points that were randomized and that had a non-missing value on the primary proximal outcome). Figure 3 shows how the analytic sample of the moderation analyses accumulated multiple sources of missingness. The reduced sample impacts the precision of the estimated interaction effects.

#### Future JITAIs in practice

If a candidate tailoring variable contains missing data during the MRT, it is possible that this variable will contain a similar amount of missing data when it is used as a tailoring variable in a future deployment of the optimized JITAI. Using the current MRT to inform how to intervene when tailoring variables are missing can be as important as identifying good tailoring variables and determining how to intervene when tailoring variables are observed.

Unfortunately, the moderation results under a complete-case analysis that excludes decision points with missing values in the candidate tailoring variable do not generate empirical knowledge about how best to intervene when tailoring variables are missing. For example, suppose participants’ recent nicotine use (e.g., participants reported smoking cigarettes recently) moderates the intervention effect; that is, prompting increases engagement with self-regulation when participants report nicotine use, but has a minimal effect when participants report no usage. If a JITAI were to be developed based on this result, prompts should probably be delivered only at decision points when participants report recent nicotine use, but not at decision points when they report no recent use. However, such findings do not inform how to act when information on participants’ recent nicotine use is missing.

We suggest extending the moderation analysis by coding the missing status of nicotine use as a separate category and modeling it as a second moderator for the effect of prompting. This allows for estimating the effect of prompting separately for the three scenarios:

- When participants report recent nicotine use,
- When they report no recent nicotine use, and
- When they do not answer the question.

Analysis like this will guide future JITAIs in using the observed values, as well as the missing status, of tailoring variables to tailor the delivery of intervention. As mentioned above, inability or refusal to answer a question is not merely a lack of certain information about a participant’s state, but also a potential indicator of useful state information in its own right (e.g., suggesting that the participant may be busy or uninterested).

## Multiple imputation in MRT

In this section, we discuss challenges pertaining to multiple imputation in MRTs and why we believe it may not always be suitable in the MRT context. Multiple imputation is a popular method for accommodating missing data in making statistical inferences (Rubin, 2004; Schafer & Graham, 2002; Van Buuren, 2018). Common implementations of this method operate under the assumption that the missing data mechanism is *missing at random* (Rubin, 2004; Schafer & Graham, 2002; Van Buuren, 2018); that is, the probability of missingness is related to observed variables. Provided that this assumption holds true and that the imputation model is correctly specified, multiple imputation removes biases caused by missing data, makes efficient use of the data that are available, and correctly adjusts standard errors to account for the increased variance due to the uncertainty of imputation.

However, to date, there are no established best practices for performing multiple imputation for the different types of variables collected in MRTs. Methodological research on multiple imputation options for longitudinal data is ongoing (see Wijesuriya et al., 2025), and future work might show how to adapt one of these methods to MRT analyses. The process of developing imputation models for MRTs is complex for several reasons. The first challenge occurs due to the intensive longitudinal structure of MRT data. One imputation approach, which follows the logic of multiple imputation for sequential multiple assignment randomized trials (Shortreed et al., 2014), suggests that missing values at time $t$ should be predicted using all data from time $1, 2, \dots , t-1,$ and $t$, but not data from time $t+1$ or later. Separate imputation models need to be built for each of the many decision points in an MRT using data up to that point.

Another challenge occurs due to the restricted randomizations. In MARS, the measurement of the proximal outcome was administered only at decision points that were eligible for randomizations. Now, suppose an analyst wants to use the proximal outcome at the previous decision point (${Y}_{t-1}$) to impute missing values of the proximal outcome at the current decision point (${Y}_{t}$). This model only makes sense among participants who are eligible for randomizations and can logically have a non-missing value at both time $t$ and $t-1$. For participants who are ineligible at time $t-1$, they do not and should not have a ${Y}_{t-1}$ value; thus, a different imputation model that does not involve using ${Y}_{t-1}$ as a predictor is needed for imputing ${Y}_{t}$. In other words, the restricted randomization results in planned missingness in MRT data, which complicates the imputation modeling.

Setting aside the technical challenges, in this paper, we suggest that imputation-based methods may not be suitable to address all sources of missing data in MRTs for all analytic purposes. Imputing missing values may not be consistent with how variables will be used in deciding how and when to intervene in future JITAIs. For example, consider candidate tailoring variables that are evaluated as potential moderators of intervention effects. A goal of the moderation analysis is to produce results that can be used to derive practically implementable JITAI decision rules. Multiplying imputing missing values in the candidate tailoring variable for the moderation analysis may not reflect how this variable would be used in practice, as a future JITAI likely will not perform imputation for missingness in tailoring variables in real time. The JITAI will probably need a separate “*if* the tailoring variable is missing, *then*…” rule to deliver an intervention option at the moments when the context information on participants is not available. To develop this rule, empirical knowledge is needed on which intervention option is the most effective when the tailoring variables are missing. This requires the moderation analysis to treat missingness in the candidate tailoring variable as a separate category and model it as a separate moderator.

In sum, while we believe that it is essential for future methodological research to provide empirically informed and standardized guidelines on how multiple imputation can be done in MRTs with various designs, we also believe that multiple imputation is not always necessary or the best approach for handling missing data in MRTs. When thinking about the implementation of JITAIs in practice, missing data about participants may represent a real, practical, and meaningful situation that the tailored intervention needs to accommodate. Correspondingly, treating missing information as a separate category in MRT analyses may provide valuable information for the development of JITAI decision rules precisely because the occurrence of missingness is not random.

## Future research

It has often been said that the best approach to handling missing data is to avoid it (Little et al., 2012). More research is needed to develop strategies to retain participants and reduce missing data via the study design (Nahum-Shani, Rabbi et al., 2021b; Rabbi et al., 2018). To borrow ideas from survey methodology, there are many considerations that go into the use of incentives (Dillman et al., 2014; Mercer et al., 2015; Singer & Ye, 2013). For example, giving a small prepaid incentive may be more effective in encouraging completion than promising a conditional incentive because it is no longer a payment that compensates for participants’ time and effort, but a token of appreciation that elicits a sense of reciprocity (Dillman et al., 2014; Mercer et al., 2015; Singer & Ye, 2013). Developing effective and efficient ways to incentivize MRT participants can be crucial for encouraging completion of research assessments. However, a distinction should be drawn between the research aspect of the MRT and the interventions embedded in the trial, as the strategies for reducing missing data may be different in the two contexts. For example, if EMAs are administered only to collect research outcomes for evaluating the efficacy of intervention options, then the use of momentary incentives may be a straightforward way to encourage participants to complete study assessments. However, if EMAs also collect information that is used as tailoring variables for intervention (e.g., self-reported stress for tailoring the content of messages), then incentivizing participants may no longer be practical and scalable when rolling out the intervention in the real world.

Another area for future research is to perform nonresponse follow-up to supplement the data collected through EMAs. There has been extensive research on the utility of conducting additional follow-up assessments targeting participants who are hard to recruit through the original data collection protocol (Couper et al., 2007; Goldberg & Sciarini, 2019; Helakorpi et al., 2015; Peytchev et al., 2009). In this perspective, to understand the mechanism of missing data and implications for study findings, additional data need to be collected on the occasions that were originally missing. One way to do this is to invite participants to complete retrospective surveys (e.g., at the end of the day) about earlier moments when EMAs were delivered. Comparing the retrospective data, observed EMA data will speak to the missing data mechanism in the EMA. Such data can also be used to fit imputation models to correct potential biases in estimated intervention effects caused by missing data. Additionally, qualitative interviews may be conducted with participants at the end of the trial to explore moments when they do not report their behaviors and states. Such qualitative data may help investigators to identify the causes of missingness and determine the best ways to intervene when real-time information about participants’ status is unavailable.

How to incorporate anticipated missing data into sample size planning is also an important area for future research. To retain a specific level of power despite missing data, one intuitive approach is to compute the needed sample size without considering missing data and then inflate the size according to the expected proportion of missing data. This is simple but has the limitation of treating the proportion of missing data as the same throughout the duration of the study. Not accounting for potential time-varying patterns in missing data may result in the estimated sample size being smaller or larger than what is actually needed. Alternatively, to account for the time-varying pattern of missing data, investigators may make use of the “expected availability” input parameter (referred to as “eligibility” in the above discussion) in the MRT-specific sample size calculators(Liao et al., 2016; Seewald et al., 2016). Specifically, the “expected availability” parameter may be treated as a composite that incorporates both the expected availability rate and the anticipated missing data rate. Since this parameter can be specified as time-varying, it provides a practical workaround for capturing changes in missing data rates over the course of the study. However, this approach may be limited if the pattern of missing data differs from the pattern of ineligibility and cannot easily be combined into one composite trend. Additionally, this approach implicitly changes the estimand from “the average proximal treatment effect when participants are eligible” to “the average proximal treatment effect when participants are eligible and provides the data to estimate this effect;” the latter is convoluted to interpret, particularly for the purpose of informing intervention optimization. Overall, we speculate that the choice between these approaches depends on one’s assumption about the longitudinal pattern of missing data (e.g., an increasing or a constant missingness rate). More research is needed to develop efficient and easy-to-use approaches for incorporating the various sources of missing data into sample size planning, while also simplifying the need for parameter inputs from substantive researchers.

In this paper, we focus on the use of MRTs to answer questions about how to best construct JITAIs. However, MRTs can also be used to answer questions about how to best construct personalizing JITAIs (pJITAIs), an emerging intervention design where the decision rules governing the JITAI are optimized as the individual experiences the intervention in real time. Specifically, pJITAIs use reinforcement learning (RL) algorithms to update JITAI decision rules based on information accrued during the intervention about the person’s responsiveness to and engagement with the intervention options (Coughlin et al., 2024; Nahum-Shani & Murphy, In Press; Nahum-Shani et al., 2024). The management of missing data in these settings is more complex and may require additional methodological considerations.

In general, the concept and use of the term “missing data” should also be clarified and expanded to reflect the nuances in its meaning. These nuances include what is missing, why it is missing, and how the missingness is related to specific research questions or practical considerations. The implications and management of missing data depend on these issues. In the statistics literature, missing data are often discussed under a theoretical assumption that a true value exists in a superpopulation but is not observed; the goal of the analysis is to make inferences using the incomplete data, aiming to approximate the estimate as if no values are missing (Schafer & Graham, 2002). However, this assumption does not apply to all forms of missingness. For example, missing data can sometimes mean that no definable true value exists (e.g., spouse’s nicotine use among individuals without a spouse), and the dataset, despite having blank cells, is actually complete (Dziak & Henry, 2017). Additionally, if the goal is to inform real-world practice where missing information is part of reality, it may not be appropriate to make assumptions about the true unobserved values in the superpopulations. Instead, missingness should be treated as a separate and meaningful category, where “no answer” can serve as an implied or partial answer.

## Conclusion

This paper articulates the various sources and distinctive implications of missing data in MRTs, which are important for MRT research for three key reasons. First, the diverse modalities and timing of data collection create multiple sources of missing data, which distinguishes MRTs from observational studies and standard randomized controlled trials. Our goal is to help future investigators better anticipate and plan for these multiple sources of missingness, so they can attain higher-quality data for addressing their scientific questions. Second, missing data in MRTs affect statistical inferences not only by making the analytic dataset incomplete in the classic sense, but also by changing the intervention and trial design. Articulating the implications of missing data will help investigators make more informed decisions about how to minimize missing data, address missing data, and interpret subsequent findings. Third, certain types of missing data in MRTs should be managed with the understanding that missingness will be an inevitable aspect in future JITAIs. Therefore, the goal of the MRT analysis is not always to recover missing values or to approximate statistical inferences as if no values are missing. In many situations, missing data may need to be conceptualized as a distinct state in its own right, based on which an intervention decision should be made in practice.

Our discussion shows that the management of missingness in different MRT variables may result in changes in the intervention, changes in scientific questions about the intervention, and changes in the estimation method for the questions. There is not necessarily a right or wrong approach for the various sources of missing data in MRTs, but future research will benefit from an awareness of these nuances and thoughtful consideration of missing data during the design of an MRT. Implications of these choices extend to the interpretation and precision of the MRT results, and the future JITAIs to be developed based on the MRT findings.

## Supplementary Information

Below is the link to the electronic supplementary material.

Supplementary file1 (DOCX 25 KB)

## Data Availability

The datasets analyzed in the current study are not publicly available but are available upon reasonable request. Because of this limitation, the analysis code is illustrated with a synthetic dataset, which allows readers to check the correctness of their implementation. Synthetic data are deposited on the GitHub page: https://github.com/ShiyuZhangGH/open-code/tree/main/MRT_missing_data_submit_code_and_data

## Code availability

Code is deposited on the GitHub page https://github.com/ShiyuZhangGH/open-code/tree/main/MRT_missing_data_submit_code_and_data
