# Supplementary material for: Missing data in microrandomized trials: Challenges and opportunities
Source: Behav Res Methods. 2025 Nov 14;57(12):344. doi: 10.3758/s13428-025-02885-y (PMC12618347; doi:10.3758/s13428-025-02885-y)
Supplement: Supplementary file 1 — Supplementary file1 (DOCX 25 KB) [file 13428_2025_2885_MOESM1_ESM.docx]

**Appendix 1**

This appendix provides step-by-step instructions using R code and artificial data to demonstrate how to do the analyses mentioned in this paper.

**Artificial data setup**

Consider a hypothetical MRT that has a similar design and collects variables similar to those of MARS (Nahum-Shani, Potter, et al., 2021). Each study day contained six decision points. A decision point is deemed eligible for randomization if, at the time, the participant is not driving or when driving information is missing (variable drive). If a decision point was eligible, one question was administered asking about the person’s cigarette availability (variable cigAvailable). If participants completed this question, they were immediately randomized to one of two intervention options; otherwise, they were randomized two minutes later. The two intervention options include 1) no prompt and 2) prompt recommending a self-regulatory strategy (variable A, meaning Action). The recommendation is tailored based on the participant’s answers to this question. One hour following each randomization, participants were invited to complete an EMA, which included items pertaining to participants’ engagement with self-regulatory strategies in the past hour (variable engage), as well as their substance use (variable recentUse). Pre-specified covariables include age and income measured at the baseline (variables age and income). All variables collected in this hypothetical MRT are summarized in Table A. We create an artificial dataset that includes 100 participants. The data are not proposed to be fully realistic, but are provided online to allow readers to practice different methods.

Missingness in the proximal outcome (engage) are specified as a function of age in the sense that older participants are more prone to missing data (i.e., missing at random). Missing data in all other variables are simulated as missing completely at random.

| Table A. Variables in the hypothetical MRT. | | |  |
| --- | --- | --- | --- |
| Variable Type | Variable Name | Values | Missing Proportion |
| Intervention indicator | A | Binary:  1 = prompt; 0 = no prompt | 0% |
| Proximal outcome | engage | Binary: 1 = engage in self-regulatory strategies in the past hour;  0 = do not engage in self-regulatory strategies in the past hour;  missing = unknown | 20% |
| Covariate | age | Continuous:  Mean = 40; standard deviation = 10 | 10% |
|  | income | Categorical:  1 = low; 2 = medium; 3 = high | 10% |
| Embedded tailoring variables that restrict randomization (i.e., eligibility) | drive | Binary:  1 = driving, thus not eligible; 0 = not driving, thus eligible; missing = unknown, regarded as eligible | 20% |
| Embedded tailoring variables that do not restrict randomization (i.e., eligibility) | cigAvailable | Binary:  1 = yes; 0 = no; missing = unknown | 20% |
| Candidate tailoring variables | recentUse | Binary:  1 = yes; 0 = no; missing = unknown | 20% |

**Instructions and R Code**

We begin by reading the artificial MRT data. This a long-format dataset with 6000 rows, corresponding to 100 participants * 60 decision points.

| all_data<- read.csv("example-MRT-data.csv") |
| --- |

**3.1. Primary Outcome.** In the main text, we describe analyses for inspecting the pattern of missingness in the proximal outcome. Here, we demonstrate a type of analysis that sheds light on whether participant characteristics are predictive of their missingness. For example, are younger participants more prone to missing data in the outcome than older participants? This analysis can be performed at the person level. The original dataset at the person-decision-point level needs to be aggregated to the person level; we make use of the group_by() and summarise() functions in dplyr package (Wickham et al., 2023).

*For each participant,* we calculate the proportion of decision points at which the outcome is observed*.* In our example, the outcome assessment is administered only if a decision point is eligible for randomization. Hence, the denominator of this proportion is the total number of decision points at which a participant is eligible for randomization: n_elig. The numerator of this proportion is the number of decision points at which a participant completes the outcome assessment: n_obs. Each participant’s proportion of observed outcome is calculated as n_elig/n_obs. In addition, we retain participant characteristics (age and income) in the newly created person-level dataset.

| dat_person<- all_data%>%  filter(drive==0 \| is.na(drive))%>%  group_by(person_id)%>%  summarise(n_elig= n(),  n_obs= sum(!is.na(engage)),  prop_obs= n_obs/n_elig,  age= first(age),  income= first(income)) |
| --- |

To examine whether participants with certain characteristics are more prone to missing data, we fit a linear regression model, relating their proportions of observed outcome to their characteristics (age and income in our example). Significant associations imply that missing data do not occur evenly among participants with different characteristics. In our example, there is a significant association between age and the proportion of observed outcome, suggesting that older participants are more prone to missing data (b = -0.008, p < 0.001). Income is not associated with the proportion of observed outcome (dummy variable 1: b = 0.013, p = 0.411; dummy variable 2: b = 0.015, p = 0.320). This is consistent with the missing at random mechanism in the artificial data, where the occurrence of missing data is simulated as a function of age.

| lm1<- lm(prop_obs~ age + factor(income), data= dat_person)  summary(lm1) |
| --- |

**3.2. Covariates.** We describe a missing indicator method in the main text as a way to handle missingness in covariates when estimating the average intervention effect. Here, we demonstrate how the missing-indicator method is implemented. Suppose that we use complete-case analysis to manage missingness in the proximal outcome by excluding rows with missingness in the outcome.

| analysis_data<- all_data%>% filter(!is.na(engage)) |
| --- |

In our example, there are two pre-specified covariates, age and income, both of which contain missing data. For the continuous variable age, the use of the missing-indicator method involves imputing a constant to the missing values (age_cimputed) and creating a binary indicator indicating missingness (age_missIndicator). We imputed the sample mean of age as the constant; other constants (e.g., 0) would be valid in this context and lead to the same main effect estimate, although this would change the intercept and standard errors. For the categorical variable income, the use of the missing-indicator method involved creating missingness as a separate category in the original variable (income_cimputed). This category will be coded as an additional dummy variable later in the analysis, which is functionally the same as creating a separate missing indicator.

| analysis_data<- analysis_data%>% mutate(  age_cimputed= case_when(is.na(age)~ mean(age, na.rm= T),  TRUE~ age),  age_missIndicator= case_when(is.na(age)~ 1,  TRUE~ 0),  income_cimputed= case_when(is.na(income)~ 0,  TRUE~ income)) |
| --- |

Proceeding to the MRT analysis, we include both the constant-imputed covariates and the missing indicators as covariates. By default, the emme()function in the MRTanalysis package does not output the coefficients of covariates, but we can set the parameter show_control_fit= TRUE to output these coefficients.

| mrt1 <- emee(  data = analysis_data,  id = "person_id",  outcome = "engage",  treatment = "A",  rand_prob = 0.5,  moderator_formula = ~ 1,  control_formula = ~ age_cimputed+ age_missIndicator+ factor(income_cimputed),  availability = "eligible")  summary(mrt1, show_control_fit= TRUE) |
| --- |

**3.3. Embedded tailoring variables that restrict randomizations.** In our example, we created 20% missingness in the eligibility information, variable drive. We assume a scenario where decision points with missing eligibility information are regarded as eligible and randomized. However, if decision points with missing eligibility information are regarded as ineligible, the analysis sample will be reduced by 20%.

**3.4. Embedded tailoring variables that do not Restrict randomizations.** In our example, the variable cigAvailable is an embedded tailoring variable that does not restrict randomization but could be used to customize messages on occasions when messages are randomly sent. We argue in the main body of the paper that missing data in this variable should be handled at the design stage (e.g., by specifying a course of action when it is unclear how to customize the message).

**3.5. Candidate tailoring variables.** In the main text, we have suggested investigating missingness in the candidate tailoring variable as a possible moderator for the intervention effect. The results will inform not only how to intervene when the tailoring variable is observed, but also how to intervene when this variable is missing. We demonstrate how the interaction analysis can be performed.

To prepare the dataset, again, suppose that we use complete-case analysis to manage missingness in the proximal outcome by excluding rows with missingness in the outcome.

| analysis_data<- all_data%>% filter(!is.na(engage)) |
| --- |

Then, we create variables that will be analyzed as potential moderators. In our example, we analyze whether substance use (recentUse) measured at the previous decision point (t-1) moderates the intervention effect at the current decision point (t). We use the lag()function (Wickham et al., 2023) to create a lagged variable (recentUse_lag1) that aligns substance use data at t-1 with intervention assignment at time t.

| analysis_data<- analysis_data%>%  group_by(person_id)%>%  mutate(recentUse_lag1= lag(recentUse))%>%  ungroup() |
| --- |

Next, because recentUse is a binary variable, creating an indicator for its missingness is equivalent to coding the missingness as a separate category. We create a new variable recentUse_lag1_cimpute, in which the value 2 is a separate category indicating missing data.

| analysis_data<- analysis_data%>%  mutate(recentUse_lag1_cimpute= case_when(is.na(recentUse_lag1)~ 2,  TRUE~ recentUse_lag1)) |
| --- |

Proceeding to the MRT analysis, we include recentUse_lag1_cimpute, a factor-type variable, as the potential moderator. With three categories (0 = no use; 1 = use; 2 = missing), recentUse_lag1_cimpute is represented by two dummy variables. Consequently, the model includes two interaction terms between these dummy variables and the randomized intervention assignment; this allows us to evaluate how the intervention effect differs across the different levels of recent substance use, including the scenario where substance use data are missing. The results show that neither recentUse (b = -0.049, p = 0.620) nor the missingness of recentUse (b = -0.048, p = 0.639) moderates the intervention effect; this is expected because we didn’t specify heterogeneous intervention effects in the artificial data.

| mrt2 <- emee(  data = analysis_data,  id = "person_id",  outcome = "engage",  treatment = "A",  rand_prob = 0.5,  moderator_formula = ~ factor(recentUse_lag1_cimpute),  control_formula = ~ factor(recentUse_lag1_cimpute),  availability = "eligible") |
| --- |

**Appendix 2**

This appendix provides a detailed introduction of the missing-indicator method (MIM), explaining why this method can be regarded as valid when analyzing randomized trials, but not when analyzing observational studies. The discussion here is grounded in the context of randomized-controlled trials.

As mentioned in the main text, the MIM refers to treating the missing values of a covariate as its own category and creating a separate indicator to denote the missingness. For example, using the MIM on a continuous variable “income” involves replacing the missing income values with a constant (such as a grand mean; the choice of the constant only affects the intercept term in the regression model used to analyze the data), creating a new binary variable to indicate the missingness (e.g., 0 for reported income and 1 for missing income), and then including both the constant-imputed income and the missing indicator as a covariate pair in subsequent analyses.

It is critical to note that the MIM is specifically valid for handling missing data in covariates for estimating the average treatment effect using randomized trial data; that is, this method *cannot* be used generally to handle missing covariates in all scenarios. Our discussion here is consistent with the work by (Groenwold et al., 2012; Kayembe et al., 2022; Sullivan et al., 2018; White & Thompson, 2005; Zhao & Ding, 2024), and is not in conflict with the expert sources (Graham, 2009; Jones, 1996; Schafer & Graham, 2002) that recommend against MIM for the management of missing data in observational data.

To illustrate why this is the case, consider a simple algebraic example. Suppose that there are three variables collected in a randomized trial—Treatment assignment (T), a covariate Income (Inc), and the experimental outcome Wellbeing (*Well*). Treatment assignment and Income are independent of each other (on average) due to randomization. Suppose Income contains missing values. We use MIM to impute zero to the missing income values (${Inc}^{0imp}$) and then create a dummy variable to indicate missing income (*MissInc*). The treatment effect can be estimated as:

$Well=\beta_{0}+\beta_{1} T+\beta_{2}{Inc}^{0imp}+\beta_{3}MissInc$ (1)

The estimated treatment effect $\beta_{1}$ is unbiased. The reason becomes clear when we write the model in a two-part form:

$Well=\left\{ \begin{aligned} \beta_{0}+\beta_{1} T+\beta_{2}Inc, if Income is observed \\ \beta_{0}+\beta_{1} T+\beta_{3}, if Income is missing \end{aligned} \right.$ (2)

This two-part model corresponds to a situation where the two models are fitted on the two sample subgroups with and without Income values, respectively: For the first subgroup with observed Income values, the treatment effect is estimated adjusting for Income. For the second subgroup with missing Income values, the treatment effect is estimated without adjusting for any covariates. Both ways of estimating treatment effects are valid and unbiased due to randomization. That is, if we assume that randomization has balanced Income across experimental arms (on average), then covariate adjustment only affects the precision (standard error) of the treatment effect, but not the bias of the treatment effect.

Intuitively, Model (1)’s coefficients can be thought of as weighted sums of the coefficients obtained from fitting the two models separately on the two sample subgroups. Hence, the treatment effect estimated in Model (1) is unbiased.

However, MIM is inappropriate in observational studies. To illustrate the reason, consider an example where three variables are all positively associated with each other—Age, Income (*Inc*), and Wellbeing (*Well*). Age and Wellbeing are fully observed, while Income contains missing values. Suppose an investigator is interested in a model in which Wellbeing is the outcome variable, and Age and Income are the predictors. Using MIM to manage missingness in Income, the model can be specified as:

$Well=\beta_{0}+\beta_{1} Age+\beta_{2}{Inc}^{0imp}+\beta_{3}MissInc$ (3)

Unfortunately, the estimated coefficients $\beta_{1}$and $\beta_{2}$ are biased. Again, the reason becomes clear when we write the model in a two-part form:

$Well=\left\{ \begin{aligned} \beta_{0}+\beta_{1} Age+\beta_{2}Inc, if Income is observed \\ \beta_{0}+\beta_{1} Age+\beta_{3}, if Income is missing \end{aligned} \right.$ (4)

This two-part model corresponds to a situation where the two models are fitted on the two sample subgroups with and without Income values, respectively: For the first subgroup with observed Income values, the model includes both Age and Income as predictors. For the second subgroup with missing Income values, only Age is modeled as a predictor. Because Age and Income are positively associated, leaving Income out of the model means that a part of the association between Income and Wellbeing is now mistakenly captured by Age. This leads to an overestimated coefficient of Age.

Intuitively, Model (3)’s coefficients can be thought of as weighted sums of the coefficients obtained from fitting the two models separately on the two sample subgroups. As the result, the coefficient of Age ($\beta_{1})$ tends to be overestimated because it is overestimated in the second subgroup with missing Income. The overestimation of $\beta_{1}$ leads to the underestimation of $\beta_{2}$.

These two toy examples demonstrate a key difference between randomized trials and observational studies. In observational studies, predictors are almost always mutually associated with each other; hence, the misspecification of any predictor can affect the coefficients of all predictors. Nonetheless, randomized trials represent a different context: Under the assumption that the randomization procedure is successful, there is independence between the treatment indicator and all covariates (in expectation, in long-term average). Hence, the specifications of missing covariates do not influence the consistency of the estimated treatment effect. To make the argument concrete, consider a twist in the above example on the observational study. If Income and Age were completely independent, leaving Income out of the model would not affect the association between Age and Wellbeing in the second part of the model among the subgroup with missing Income. As the result, the coefficients of Age and Income in Model (3) would be unbiased. Now, replacing Age in this example with a treatment indicator; this explains why the use of the missing indicators does not invalidate the estimate of the treatment effect.
